# Supplementary material for: Characterization of a Novel Rice Dynamic Narrow-Rolled Leaf Mutant with Deficiencies in Aromatic Amino Acids
Source: Int J Mol Sci. 2020 Feb 23;21(4):1521. doi: 10.3390/ijms21041521 (PMC7073152; doi:10.3390/ijms21041521)
Supplement: Supplementary file 1 [file ijms-21-01521-s001.pdf]

Table S1 Markers used for fine mapping of *DNRL1*

Primer	Forward sequence	Reverse sequence
S1	5'-TTCGATGTGACATGGTGA-3'	5'-TCTTCGTGCGAGTAGGTT -3'
S5	5'-GGTTGGTACGCCTACGTC-3'	5'-GGGGCTTTTGATGATGGG-3'
S11	5'-AGGGTCGGTGGGGTAGGT-3'	5'-GAGGAGGCCGGTGAAATT-3'
RM21981	5'-TTAATCCAGCTCCTCTGACTTTGG-3'	5'-TGGCGGCTTAGGAGTGTTTATAGG-3'
RM21985	5'-GTCGGTATCTGCGCGTACTTGG-3'	5'-GGCCCTGTAATGAGACGACAAGC-3'
RM21989	5'-AGGTGCAGGTGAGGTGACTCC-3'	5'-CACCTTCCTTCCATTCGACTCC-3'
RM18	5'-TTCCCTCTCATGAGCTCCAT-3'	5'-GAGTGCCTGGCGCTGTAC-3'
RM8261	5'-GACGACTGGATGGTACGAC-3'	5'-TGCTTCTCCTGCAAACAC-3'

Table S2 Performance of agronomic traits in IR64, *dnrl1*, complementation and overexpression lines

Material	Plant height(cm)	No.of panicles	Seed-setting rate (%)	1000-grain weight(g)
IR64	109.1 ±1.2	22.7 ±2.3	65.8 ±3.3	26.3 ±0.3
dnrl1	83.1 ±2.8**	17.3 ±2.5	67.5 ±0.4	22.8 ±0.7**
dnrl1-c1	84.0 ±1.1**	19.3 ±2.9	41.5 ±6.4*	22.1 ±0.3**
dnrl1-c3	81.7 ±2.1**	21.7 ±1.5	67.2 ±4.3	22.2 ±0.3**
dnrl1-ox3	82.1 ±0.9**	21.3 ±1.5	52 ±1.7*	22.1 ±0.2**
dnrl1-ox7	82.6 ±1.5**	21.7 ±1.5	55.7 ±3.9	22.8 ±0.7*

Values are means ± SD (n=3), * indicates significance at $P \leq 0.05$;** indicates significance at $P \leq 0.01$; dnrl1-c1 and dnrl1-c3 are complementation lines; dnrl1-ox3 and dnrl1-ox7 are overexpression lines.

Table S3. List of genes used for qRT- PCR analysis

Gene	Forward primer (5'–3')	Reverse primer (5'–3')	Accession No.
OsCAO1	GATCCATACCCGATCGACAT	CGAGAGACATCCGGTAGAGC	J013116K15
OsRpoTp	ATTTCCTCCCTGTCCTTC	TGACTTGCTGCGTATGTG	AK058530
HEMA1	CGCTATTTCTGATGCTATGGGT	TCTTGGGTGATGATTGTTTGG	J013000F15
OsPORA	TGTACTGGAGCTGGAACAACAA	GAGCACAGCAAAATCCTAGACG	AK065236
PPR1	CTAAGACCGAATGACAAATGC	GCACTGCCAACAAGAATACC	AY584749
psaA	GCGAGCAAATAAAACACCTTTC	GTACCAGCTTAACGTGGGGAG	AAS46121
psbA	CCCTCATTAGCAGATTCGTTTT	ATGATTGTATTCCAGGCAGAGC	AAS46104
rpoA	ACGGAAGTCAGAAAGAAC	TACGAGAAGCCTCATAAA	NC_001320
SPP	CGGAGAGGAAACATAATGAC	ATAGGCATTTGTCTTTGTCTC	AK066566
YGL1	AACCTTACCGTCCTATTCCTT	CCATACATCTAACAGAGCACCC	EF432576
OscpSRP54	AGATGTGAGTCTCCCAGT	TCAGATACTTCTCCACCC	AY050999
DNRL1	CACAGCGAACAAGGAGACAGG	AGAGGAGGCACTCATGGGATG	AB122082.1
NAL1	TTGGTATGGAATCTATGCTGGAA	TGAGGCTATTGAGCGGACACT	EU093963
NRL1	ACCACAACCTCGTCTTCTTCG	TCTTCTTGCCCATGCTCTTCT	AK242601
OsCOW1	TAGGTTTCTACTCCTCGTTGC	CTTGATGTTTCCCGATTTGAT	AK072466
TDD1	TTTTGGTGTCTGCATGGGTCT	CTCCTCATCGTAGCGAACTGG	AK107879
ubq	GCTCCGTGGCGGTATCAT	CGGCAGTTGACAGCCCTAG	AF184280
LYL1	ACGAGTACATCGGCATGGTC	TGTAGTGCACCACGTACGAC	KF305678
OsCAO2	AGACTGTTGCAAGACGGAGG	TTGTTCACAAAACGGGACGC	AK063367
OsChlD	CATAGCAATGGCCCGCAATC	TACGTCCAACATCACCGCTC	AK072463
OsPORB	GGGTGTACTGGAGCTGGAAC	ACTGATCGTGATCGGCCAAG	AK068143
YL1	AACTCTGCCGGTGGATTCAT	GGAGCGAGCCCATTGTCATA	AK061136
YGL8	GCAGTTCCTCAACGACTGGA	TGTTGTGCGGTTGGTCTCAA	AK059435

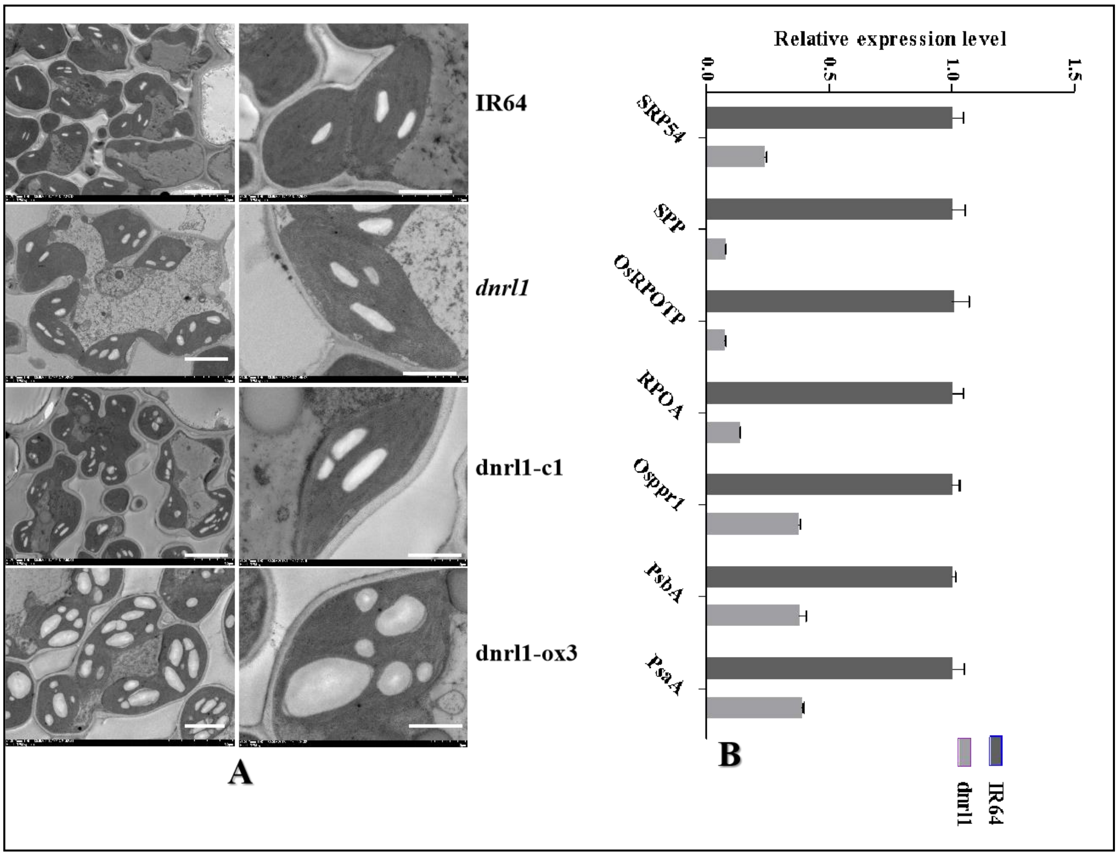

Figure S1. Ultrastructure of chloroplast and qRT-PCR analysis of photosynthesis-related genes

A, Chloroplast ultrastructure of IR64, *dnrl1*, dnrl1-c1 and dnrl1-ox3, for the left column bar=50 um, for the right column bar=20um; B, qRT-PCR analysis of 7 photosynthesis- related genes .
